# Building the foundations for an organized population-based cervical cancer screening program with primary HPV self-sampling in Catalonia, Spain: findings from a pilot implementation study

**DOI:** 10.3389/fmed.2025.1580665

**Published:** 2025-08-04

**Authors:** Paula Peremiquel-Trillas, Esther Roura, Valentina Rangel-Sarmiento, Francisca Morey, Rebeca Font, Maite Carvajal, Clàudia Robles, Raquel Ibáñez, Lara Pijuan, Lourdes Tamarit, Dolça Cortasa, Daniel Fernández, Josep Alfons Espinàs, Laia Bruni, on behalf of the Catalan Cervical Cancer Screening Program working group

**Affiliations:** ^1^Cancer Epidemiology Research Program, Catalan Institute of Oncology – ICO, Bellvitge Biomedical Research Institute – IDIBELL, Barcelona, Spain; ^2^Faculty of Medicine and Health Sciences, University of Barcelona, Barcelona, Spain; ^3^Faculty of Nursing and Health Sciences, University of Barcelona, Barcelona, Spain; ^4^Consortium for Biomedical Research in Epidemiology and Public Health – CIBERESP, Carlos III Institute of Health, Madrid, Spain; ^5^Catalan Cancer Plan, Department of Health, Barcelona, Spain; ^6^Department of Pathology, Bellvitge University Hospital, Barcelona, Spain; ^7^Barcelona Metropolitan South Health Region, Catalan Health Service, Barcelona, Spain; ^8^Catalan Institute of Oncology – ICO, Barcelona, Spain

**Keywords:** Uterine cervical neoplasms, early detection of cancer, mass screening, human papillomavirus viruses

## Abstract

**Introduction:**

As part of the transition from opportunistic cytology-based screening to an organized, population-based HPV screening program, Catalonia, Spain, launched an implementation pilot in 2021.

**Methods:**

The pilot combined home-based HPV self-sampling with pharmacy-based distribution, coordinated by a screening office using an SMS-based invitation and reminder system, alongside structured follow-up of HPV-positive cases by midwives.

**Results:**

From July 2021 to December 2023, 6,355 women seeking cervical cancer screening were invited to participate in HPV self-sampling via SMS, with high participation (80.9%). Among HPV-positive women (11.8%), compliance with triage cytology was high (98.7%), as with colposcopy referrals when indicated (97.2%). CIN2+ detection rates (3.6% overall, 13.1% in HPV-16 positive) aligned with international studies, reinforcing the value of genotype-specific risk stratification and risk-adapted follow-up pathways in our setting. This organized approach facilitated timely case management and demonstrated the feasibility, acceptability, and effectiveness of the model.

**Discussion:**

While conducted in an opportunistic screening context with a relatively short follow-up time, these findings support HPV self-sampling as an effective primary screening strategy, including women who regularly attend cervical cancer screening, and provide key insights for its scalability within a population-based program, which began its pilot phase in 2024 and is set for full implementation in 2025.

## Introduction

Human papillomavirus (HPV) self-sampling is increasingly recognized as a primary screening method in well-established cervical cancer screening programs worldwide. A growing number of countries include self-sampling within their official screening guidelines or are evaluating its use in pilot projects (1). In 2022, the European Commission updated its screening recommendations, advocating for the use of only clinically validated HPV assays as the preferred method for women aged 30 to 65, with screening intervals of at least 5 years (2). The updated recommendations also emphasize the provision of self-sampling kits for cervical cancer screening, particularly targeting women who do not participate regularly in screening programs. Aligned with this approach, the EU aims to ensure that by 2025, 90% of the eligible population is offered screening for breast, cervical, and colorectal cancers (3).

During the past decade, HPV self-sampling has emerged as a promising strategy to improve screening participation, particularly among non-attenders, including women from rural areas and racial, ethnic, sexual, and gender minorities (4, 5). By improving accessibility in hard-to-reach populations, self-sampling increases the capacity to reach individuals at higher risk of cervical cancer (6). Research shows that both regular and non-attenders experience less shame, anxiety, and discomfort with self-sampling compared to clinician-based screening, making it a well-accepted alternative (4, 7). HPV self-sampling may help overcome structural barriers to screening participation, such as social class, gender, education, income, and ethnicity, thereby promoting more equitable screening (8, 9). Combined with its comparable clinical accuracy to clinician-collected samples using HPV assays with PCR amplification (10–12), these advantages reinforce self-sampling’s potential to facilitate participation within organized screening programs. However, most supporting evidence for self-sampling use in cervical cancer screening comes from studies in hard-to-reach populations, leaving a significant gap in data on its use in routine screening populations (10, 13, 14).

In 2021, the Catalan Health Department launched an implementation pilot to evaluate HPV self-sampling as primary sample collection method within its opportunistic screening program. A previous clinical trial conducted among women attending public cervical cancer screening services in the region demonstrated high acceptability of home-based HPV self-sampling, with 75.5% of women returning the self-sampling kit when offered by their healthcare provider (15). Building on these findings, and in response to the COVID-19 pandemic, which severely disrupted cancer screening programs, there was a recognized need to rethink screening strategies and adopt alternative approaches to maintain coverage while reducing reliance on in-person healthcare visits (16–18). This implementation pilot aimed to provide critical insights to assess feasibility, acceptability, and sustainability, as well as operational requirements before upscale of a new organized HPV-based screening program.

Transitioning from opportunistic to organized, population-based cervical cancer screening presents significant challenges. Experience from several European countries shows that this shift requires restructuring service delivery, enhanced coordination, and the establishment of robust quality assurance mechanisms. In this context, piloting is essential to validate screening circuits and assess key operational components, such as governance, quality assurance, information systems, and monitoring, all needed to align with international best practices for organized screening programs (19). This study contributes to that evidence by summarizing the findings from the implementation pilot conducted from 2021 to 2023 in Catalonia. This opportunistic pilot supported further piloting of the population-based approach with individual invitations in 2024, followed by scale-up in 2025 to nearby areas, with full implementation across the entire Catalan region planned by 2029.

## Materials and methods

### Setting

In Spain, healthcare competencies are fully decentralized, with regional governments overseeing healthcare services. In Catalonia, the Catalan Health Department holds sole authority over decisions on cancer screening. Within this framework, the implementation pilot started in 2021 in some municipalities of the southern metropolitan area of Barcelona.

The pilot was first launched in El Prat de Llobregat municipality in July 2021, targeting 16,898 eligible women aged 30 to 65 years. In June 2022, the program was expanded to the Baix Llobregat-Litoral area, covering the municipalities of Begues, Botigues de Sitges, Castelldefels, Gavà, Sant Climent de Llobregat, and Viladecans, with a total eligible population of 53,340 women aged 30–65 years. These two areas correspond to two ASSIRs (Sexual and Reproductive Health Care Units), which are gynecologic primary care centers integrated within primary and specialized healthcare services.

ASSIRs provide comprehensive sexual and reproductive health services, including cervical cancer prevention and related gynecological care. Each ASSIR is staffed by midwives, obstetrician-gynecologists, nurses, psychologists, and administrative staff, ensuring a multidisciplinary approach to patient care.

Before the transition to a population-based cervical cancer screening, ASSIRs have served as the main access point for women within the opportunistic cervical cancer screening model in Catalonia. Every woman has a designated reference ASSIR and can freely schedule an appointment for cervical cancer screening. During the visit, a midwife collects a cervical sample for testing, and if the result is positive, the woman is referred to a gynecologist for further evaluation and management. The entire process follows standardized protocols, ensuring consistency and quality (20).

### Participants

The inclusion criteria for participation in the cervical cancer screening program with self-sampling in Catalonia include being aged 30 to 65, or older than 65 with a history of treatment for high-grade squamous intraepithelial lesion/cervical intraepithelial neoplasia grade 2 or higher (HSIL/CIN2+) within the past 25 years. The exclusion criteria include: residing outside the designated territories for the self-sampling implementation pilot (as described in the *Setting* section, in *Methods*); absence of a cervix due to a cause unrelated to HPV (e.g., hysterectomy for benign or malignant disease unrelated to HPV, trachelectomy, congenital cervical aplasia, or being a transgender women); presence of gynecological symptoms (such as abnormal bleeding, dyspareunia, or pelvic pain); being under ongoing follow-up for cervical pathology; having had a recent screening (cytology within the past 3 years or HPV testing within the past 5 years); being pregnant (second or third trimester) or postpartum; and having a physical or mental disability that prevents sample collection.

### Screening process

**Figure 1:**
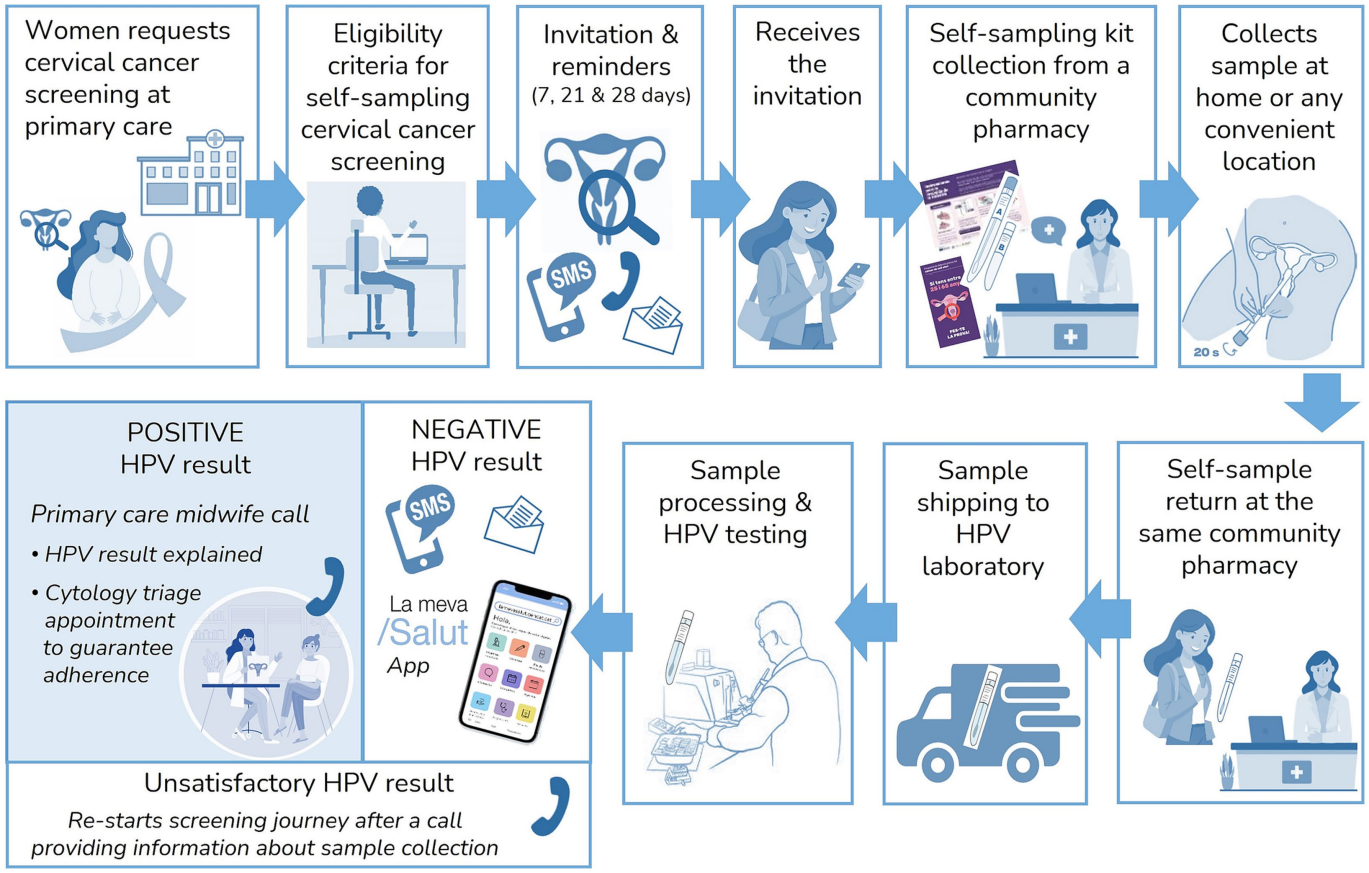
Screening process within the implementation pilot.

The screening process and adaptations made for the implementation pilot are shown in Figure 1.

Women who request cervical cancer screening at their primary care centers or gynecologic primary care centers are referred to the cervical cancer screening program to assess their eligibility for HPV self-sampling. Eligible women receive an invitation to participate in the screening program via telephone call followed by a short message service (SMS) or directly via SMS. If a telephone number is not available in the National Health System database, they are invited by letter. The SMS and letter include brief information about the screening program, the HPV test, and home-based self-sampling, along with a link^1^ directing women to the official Health Department website,^2^ where detailed information on the screening process is available. Following the initial invitation, additional reminders are sent using the same invitation method (SMS or letter) on days 7 and 21. A follow-up phone call was made on day 28 during the pilot, given that women participating in the implementation pilot demanded screening voluntarily (opportunistic program), to reinforce the importance of screening, educate women on the novel sample collection method, resolve doubts and gather the reasons for their non-participation. Reminders were only sent to women who had not participated at each stage. Those who declined self-sampling were offered an appointment for a clinician-collected HPV test in primary centers.

Pharmacies serve as distribution points for self-sampling devices. Each participant collects a kit containing the self-sampling device (FLOQSwabs^®^, Copan, Italy), a printed instruction sheet outlining the sample collection process,^3^ and an informational brochure on cervical cancer prevention.^4^ Upon kit collection, the pharmacist provides a brief explanation of how to use the self-sampling device and addresses any participant questions. When returning the sample, the pharmacists visually assess its quality, ensuring it is free of visible blood, properly sealed, and undamaged, and collected within the past 7 days (20). For sample transport and analysis, the existing shipping logistics used in the colorectal cancer screening program are utilized. Samples are dispatched daily to the laboratory for analysis (as described in the *Sample processing and HPV testing* section, in *Methods*). Samples should be processed within a two-week period from arrival at the laboratory and at a maximum time of 4 weeks after the return date of the sample to the pharmacy, as established in the screening protocol (20). HPV results are delivered to the cervical cancer screening office for participant notification and case management.

**Figure 2:**
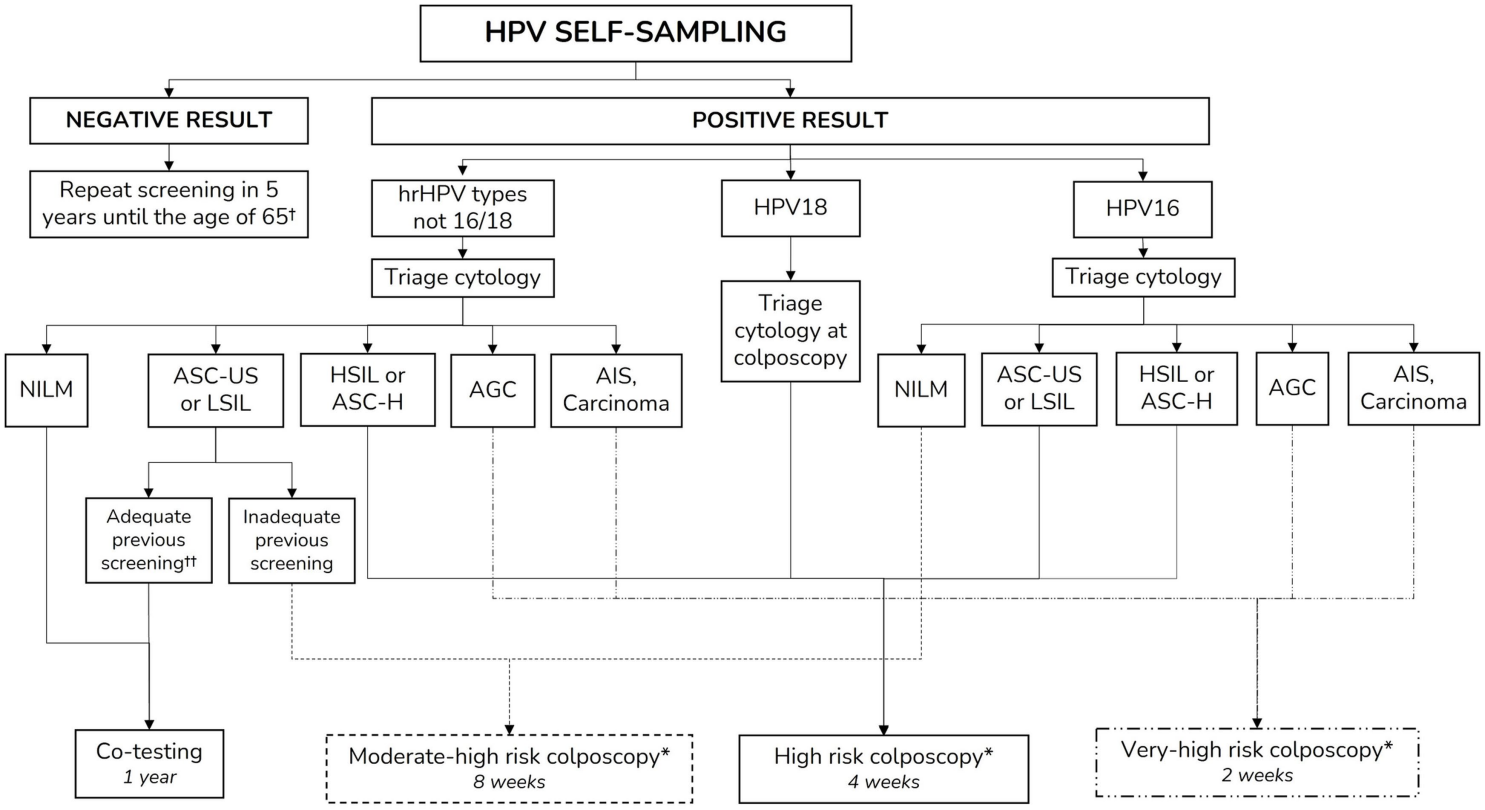
Clinical management based on HPV self-sampling test and triage cytology results. AGC, atypical glandular cells; AIS, adenocarcinoma *in situ*; ASC-H, atypical squamous cells, cannot exclude high-grade squamous intraepithelial lesion; ASC-US, atypical squamous cells of undetermined significance; HPV, Human papillomavirus; hrHPV, High-risk HPV; HSIL, High-grade squamous intraepithelial lesion; LSIL, Low-grade squamous intraepithelial lesion; NILM, Negative for intraepithelial lesion or malignancy. ^†^Or after 25 years of follow-up despite the age in case of HSIL/CIN2+ lesion treatment (19). ^††^Adequate previous screening is defined if previous negative screening with cytology within the last 3 years or with HPV testing within the previous 5 years (19). ^*^Colposcopy risk differentiation is based on the immediate risk of high-grade squamous intraepithelial lesion/cervical intraepithelial neoplasia grade 3 or higher (HSIL/CIN3+) as defined in the Catalan cervical cancer screening protocol (19).

Negative HPV results are communicated via SMS or letter (depending on the original invitation method used), directing women to access their results through the official Health Department App,^5^ where a detailed screening report specifies their results and the recommended interval for the next screening test (5 years). Unsatisfactory samples due to insufficient material are reported to women via telephone call, re-inviting them to collect a new self-sampling kit, following the same procedure as the initial invitation. If a second unsatisfactory result is obtained, women are referred for a clinician-collected sample. Women with positive HPV results are scheduled for a telephone consultation with a midwife within one to two working days. During this consultation, the midwife informs the participant of the results, clarifies doubts, and schedules further tests, such as triage cytology. The cervical cancer screening office ensures follow-up throughout the entire episode to guarantee appropriate management according to established clinical algorithms and time frames. In cases where women are lost to follow-up or a required procedure is not completed, the screening office contacts the responsible clinicians, and an educational e-mail is sent with guidance on the screening algorithms to facilitate adherence to protocols. Details of screening results management, follow-up and diagnostic procedures are outlined in Figure 2. The definitions used in the screening process are outlined in Table 1.

**Table 1:** Definitions of screening participation categories.

Eligible women	Women who *meet the inclusion criteria* for participation in HPV self-sampling cervical cancer screening.
Invited women	Eligible women who *are invited* (SMS or letter) to participate in HPV self-sampling cervical cancer screening.
Women who collects self-sampling at a pharmacy	Invited women who *collect* the HPV self-sampling kit from the pharmacy, accepting the invitation to participate in HPV self-sampling screening.
Self-sampling screening participants	Invited women who *collect* the HPV self-sampling kit from the pharmacy and *return* the sample to the pharmacy.
Self-sampling rejection	Invited women who *do not collect* the HPV self-sampling kit from the pharmacy.
Non-participating women with acceptance	Invited women who *collect* the HPV self-sampling kit from the pharmacy but *do not return* their sample.

The entire screening process is managed by the cervical cancer screening office at the Catalan Institute of Oncology, which oversees eligibility assessment, invitation and reminders, results management, quality assurance, and program evaluation. All screening data is registered in a unified screening registry within the Catalan Health Information system.

### Sample processing and HPV testing

All screening samples are analyzed at the laboratory of Bellvitge University Hospital. Upon arrival, dry swabs were resuspended in 5 mL of PreservCyt™ Solution (Hologic^®^, Marlborough, Massachusetts, USA). HPV detection is performed using the Cobas^®^4,800 PCR assay (Roche Diagnostics, Basel, Switzerland), which identifies HPV16, HPV18 and a pooled group of 12 other high-risk HPV (hrHPV) genotypes (31, 33, 35, 39, 45, 51, 52, 56, 58, 59, 66, and 68). To ensure sample adequacy and minimize false-negative results, the presence of human DNA is verified by detecting the beta-globin gene; samples that do not meet this criterion are classified as unsatisfactory.

### Data sources

Multiple data sources are used for the cervical cancer screening registry, to assess eligibility and evaluate follow-up. The target population was identified using data from the central registry of publicly insured individuals in Catalonia. Further information was obtained from the shared Medical History of Catalonia, which integrated health information from all the public healthcare centers in the region. All information from different data sources is compiled in the cervical cancer screening registry. Given that multiple municipalities with varying socioeconomic levels participated in the implementation pilot, the MEDEA index was used as a proxy for socioeconomic deprivation as it is the most used index to assess deprivation’s impact on health in our region (21–24). The MEDEA index reports deprivation for urban and rural areas, separately, establishing 4 levels of deprivation in urban settings (1U, 2U, 3U, and 4U, which correspond to least, moderately, highly and most deprived urban areas) and 2 levels in rural areas (1R and 2R, corresponding to semirural and semiurban, respectively) (23, 24). The MEDEA index is calculated using the following socio-economic information: unemployment rates, manual workers, illiterate adults and school leavers before age 16. Further details on the MEDEA index can be found elsewhere (21, 23, 24).

### Statistical analysis

Descriptive statistics were used to summarize participation and acceptance rates, as well as screening results. Categorical variables were presented as absolute frequencies and proportions. Continuous variables were categorized. Time periods were reported as medians with interquartile ranges (IQRs) due to their non-normal distribution. Differences between groups were assessed using the Chi-Square test for categorical variables and the Fisher’s Exact test when there are very low expected frequencies in the cells (<5), and the Mann–Whitney U test for non-normally distributed continuous variables. The Kolmogorov–Smirnov statistic test was performed to compare time to accept and time to participate between territories. Statistical significance was set at *p-value < 0.05*. All statistical analyses were conducted using R software (R version 4.4.1; R Core Team) through the RStudio integrated development environment (version 2024.04.2 Build 764; Posit Software, PBC) (25).

### Reporting guidelines

This study follows the RECORD guidelines (26) for the transparent reporting of observational studies using routinely collected health data. The completed RECORD is provided in Supplementary Table 1.

### Ethical approval and data protection

This study was conducted in the context of approval by the Research Ethics Committee of the Hospital Universitari de Bellvitge for activities derived from cervical cancer screening (PR271/11). It was carried out in compliance with Regulation (EU) 2016/679 of the European Parliament and of the Council, of April 27, 2016, on the protection of individuals regarding the processing of personal data. Although formal written consent was waived, participants were informed via SMS and invitation letters about the nature and purpose of the program, including the use of their screening data and samples for research. Consent was considered implied upon their agreement to participate, in accordance with ethical best practices.

## Results

### Study population and participation

**Figure 3:**
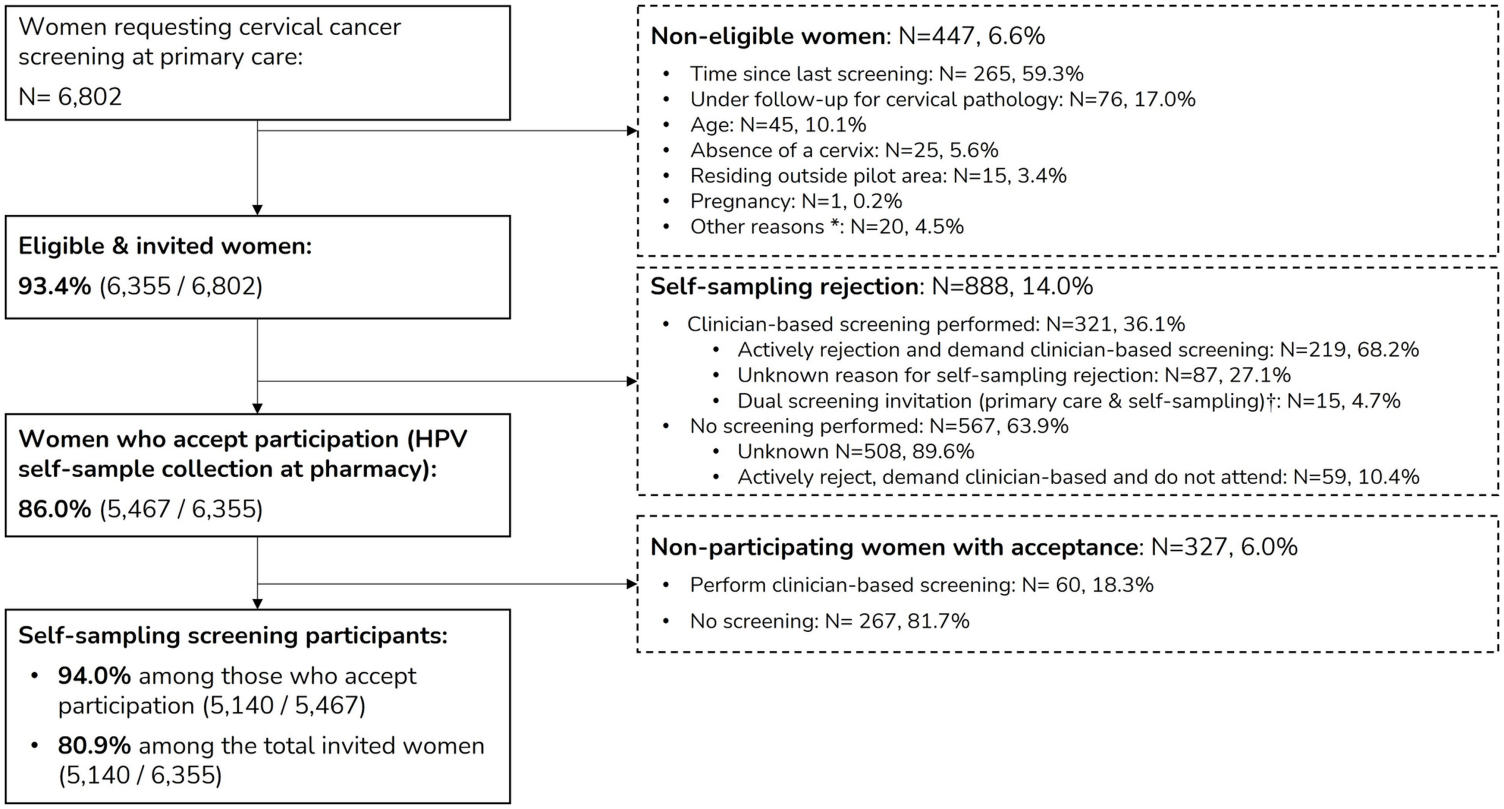
Participation flowchart. Definitions of screening participation categories can be found in Table 1. *Includes three HIV-positive women referred to primary care for clinician-collected sample due to other ongoing follow-ups, 14 women with physical disabilities and three women unable to read or understand self-sampling instructions. ^†^At the start of the pilot, due to technical issues, 15 women were mistakenly invited to self-sampling screening while simultaneously scheduled for a gynecologic primary care visit. As a result, these women had their screening samples collected during the scheduled visit, thus not participating in HPV self-sampling screening despite receiving an invitation.

From July 2021 to December 2023, 6,802 women requested cervical cancer screening in their primary gynecologic care centers (ASSIR), Of these, 6,355 (93.4%) met the eligibility criteria and were subsequently invited to participate in self-sampling. Among them, 5,467 women (86.0%) accepted the invitation and collected a self-sampling kit from pharmacies. A total of 5,140 women (94.0% of those who collected the self-sampling kit and 80.9% among the total invited) returned their self-collected samples, completing the self-sampling screening process. Women who declined self-sampling were offered the option of clinician-collected sampling, and 380 women (6%) opted for an in-person visit for sample collection by a healthcare professional. Figure 3 shows the participant flowchart, from eligibility assessment to pilot participation.

### Self-sampling participation

The sociodemographic characteristics of invited women and self-sampling screening participants are summarized in Table 2.

**Table 2:** Sociodemographic characteristics of invited and participation status.

	Invited women		Self-sampling participants		Participation (%)^2^	*p*-value^3^
	*N*	%^1^	*N*	%^1^		
Total	6,355	100.0	5,140	100.0	80.9	
Age groups						<0.001
30–34 years	779	12.3	561	10.9	72.0	
35–39 years	910	14.3	714	13.9	78.5	
40–44 years	1,143	18.0	911	17.7	79.7	
45–49 years	1,249	19.7	1,031	20.1	82.5	
50–54 years	939	14.8	774	15.1	82.4	
55–59 years	694	10.9	596	11.6	85.9	
60–65 years	641	10.1	553	10.8	86.3	
Medea Index^4^						<0.001
Semirural areas (1R)	108	1.7	84	1.6	77.8	
Least deprived urban areas (1U)	416	6.5	305	5.9	73.3	
Moderately deprived urban areas (2U)	–	–	–	–	–	
Highly deprived urban areas (3U)	3,731	58.7	2,982	58.0	79.9	
Most deprived urban areas (4U)	2,100	33.0	1,769	34.4	84.2	
ASSIR Region						<0.001
El Prat de Llobregat	2,749	43.3	2,324	45.2	84.5	
Baix Llobregat-Litoral	3,606	56.7	2,816	54.8	78.1	

1 Percentages correspond to column percentages.

2 Percentages calculated comparing participants among the total invited; corresponds to row percentage.

3 *p*-value resulting from the comparison between participants and non-participants.

4 MEDEA Index for the participating municipalities included. No 2R or 2U areas were participating in the implementation pilot. *p*-value was calculated only comparing urban settings (1U, 3U, 4U).

The median age of eligible women asking for screening was 46 years (interquartile range [IQR]: 39–54 years). Self-sampling participation was significantly higher among older age groups compared to younger ones ranging from 72% among women aged 30–34 years to 86.3% among those aged 60–65 years. Self-sampling participation varied significantly according to the MEDEA index of socioeconomic deprivation. Women living in the most deprived urban areas (4U) showed the highest participation (84.2%), while the lowest (73.3%) was observed among women living in the least deprived areas (1U) areas (Table 2). Additionally, the same participation gradient by age was observed across highly and most deprived urban areas (3U and 4U), with younger women showing lower participation rates compared to older women (Supplementary Table 2). Women who had previously participated in the cervical cancer screening program were more likely to participate in self-sampling compared to those whose screening history is unknown (82.3% vs. 74.4%) (*data not shown*).

### Time from invitation, self-sampling kit collection and sample return

**Figure 4:**
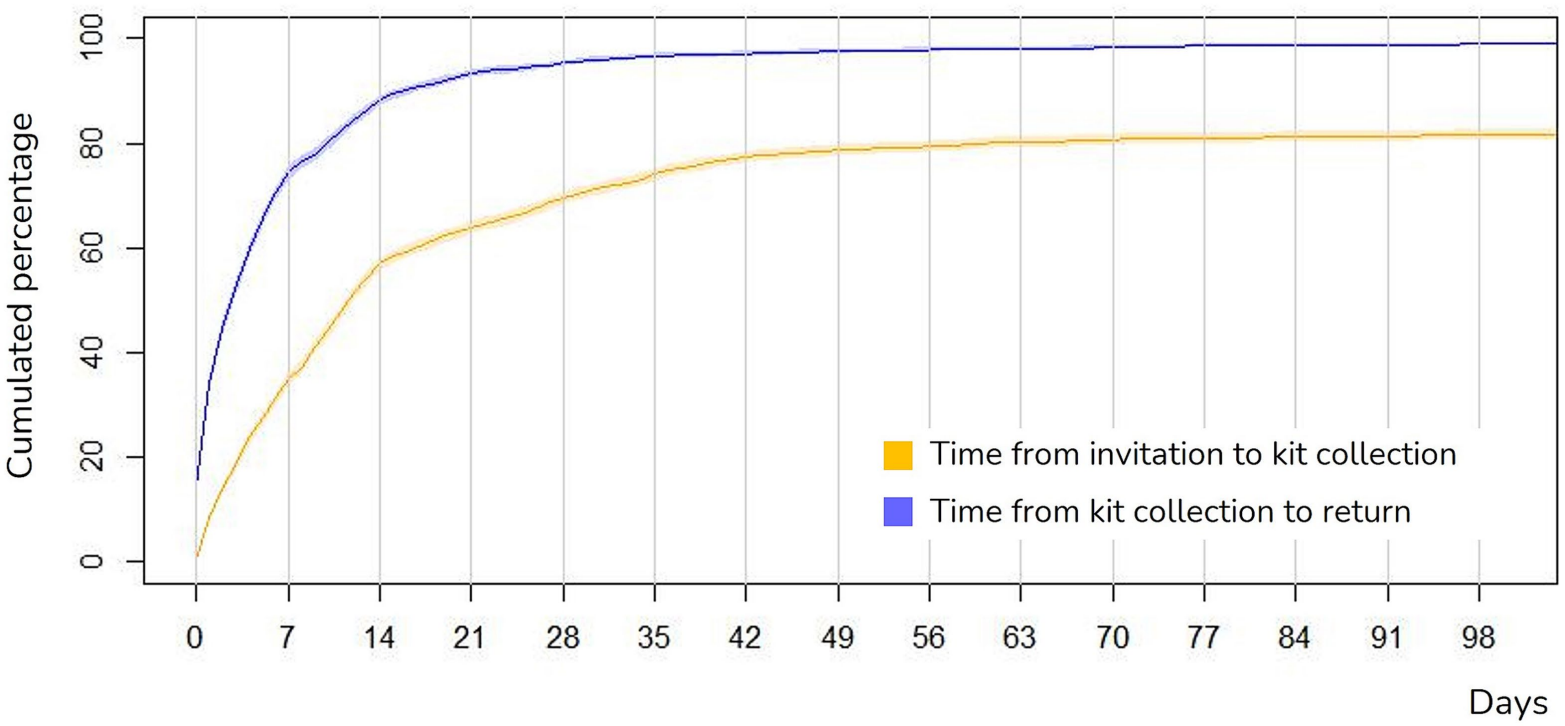
Time from invitation, self-sampling kit collection and return. Figure truncated at 100 days of follow-up, with 97.5% of women having accepted and 98.9% of women having returned the self-sample. Reminders were sent on days +7, +21, and +28.

Figure 4 illustrates the cumulative percentage of participation over time, showing the number of days from invitation to HPV self-sampling kit collection at the pharmacy as well as the time from kit collection to return. The median time from receiving the SMS invitation to collecting the self-sampling device at the pharmacy was 10 days (IQR: 4–20 days). The median time between collection and sample return was 3 days (IQR: 1–8 days).

### Participation reminders

Immediately after receiving the invitation SMS, 37.9% of women participated in the pilot. The first reminder, sent 7 days after the invitation, raised participation to 65.6%. After the second reminder, at 21 days, it further increased to 76.6%, reaching a peak of 80.9% following the third reminder (28 days). On average, the number of reminders per participant woman was 2.9, including reminders to participate as well as those to return the sample after collection.

### Turnaround times for sample processing and testing

The median time between sample return registration at the pharmacy and its arrival at the laboratory was 3 days (IQR: 2–5 days), varying slightly depending on the pharmacy and the pharmaceutical distributor. By day 7 after sample return, 89.2% of samples had already arrived at the laboratory, and by day 14, over 98.0% had been received. The median time from the sample’s arrival at the laboratory to result availability was 3 days (IQR: 1–5 days). Nearly all test results (99.7%) were available within 3 weeks of sample arrival, aligning with protocol requirements, only the results of 17 samples were reported beyond 21 days. Globally between sample return to the pharmacy and the availability of test results, the median time was 8 days (IQR: 6–12 days), and by day 21, 97.0% of women had already received their screening results.

### Repeated self-sampling collection and testing

A total of 59 women had to collect two self-sampling devices due loss of the sample during screening process (*N* = 25, 42.4%), insufficient sample (*N* = 19, 32.2%), suboptimal sample conditions (*N* = 2, 3.4%), unknown reasons/not reported (*N* = 13, 22.0%). Among these women, 53 received a valid test result after the second sample collection, two had an invalid/poor-quality result twice and were referred to a midwife for sample-collection, and four women had not yet returned their second screening sample to the pharmacy at the time of data analysis.

### HPV screening results

**Figure 5:**
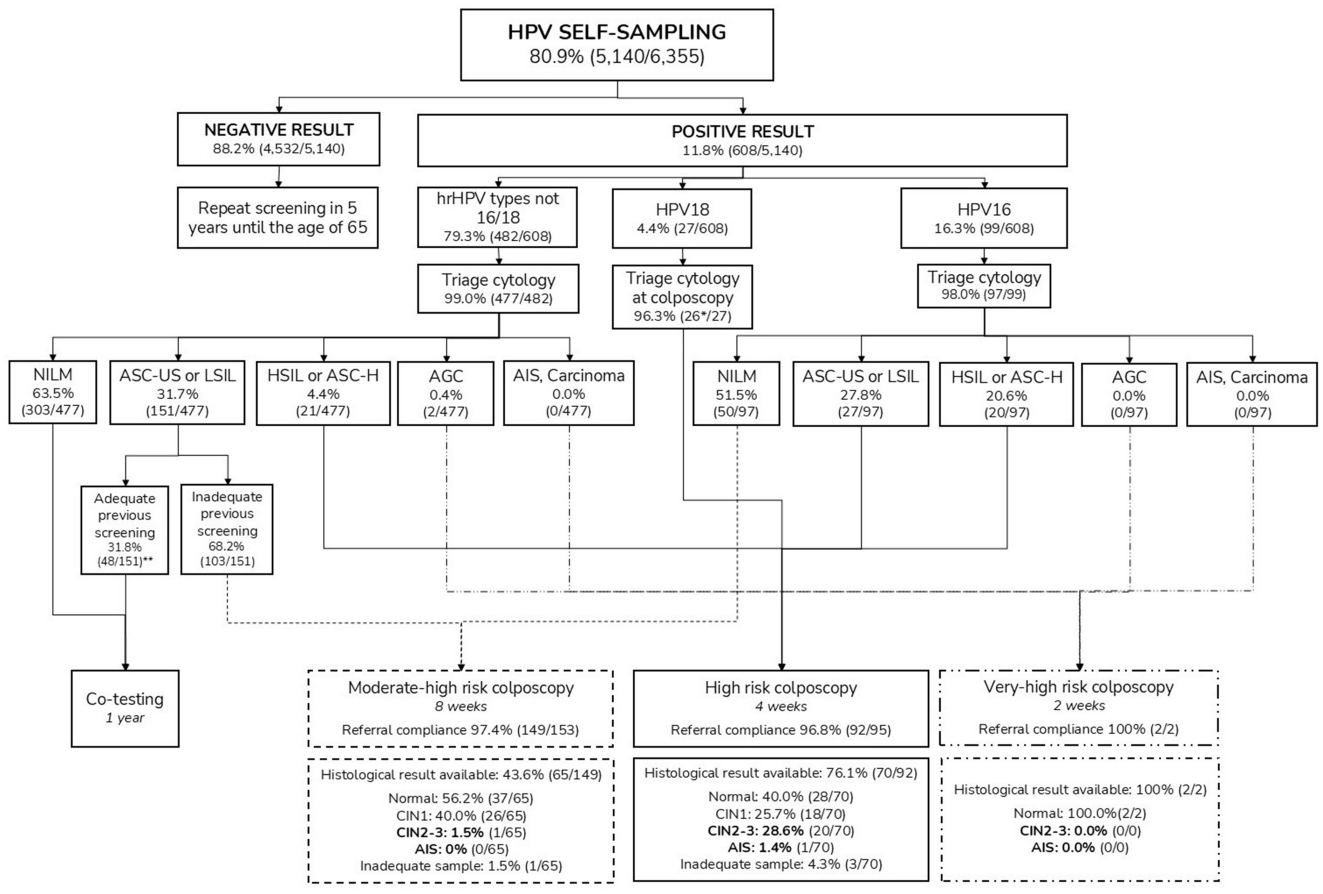
Clinical results after HPV self-sampling. AGC, atypical glandular cells; AIS, adenocarcinoma *in situ*; ASC-H, atypical squamous cells, cannot exclude high-grade squamous intraepithelial lesion; ASC-US, atypical squamous cells of undetermined significance; HPV, Human papillomavirus; hrHPV, High-risk HPV; HSIL, High-grade squamous intraepithelial lesion; LSIL, Low-grade squamous intraepithelial lesion; NILM, Negative for intraepithelial lesion or malignancy. *One woman underwent colposcopy, but triage cytology was not performed. **Ten women were referred for colposcopy and biopsy, not following the protocol recommendations (co-testing after 1 year).

Among the 5,140 self-samples processed, 608 tested positive for HPV, resulting in an overall positivity of 11.8% (Figure 5). The most frequent result was hr-HPV other than HPV16/HPV18, accounting for 79.3% (*N* = 482) of positive results (Figure 5). Positivity decreased with age, with the highest positivity rate (21.7%) observed in the 30–34 age group. The same HPV positivity gradient by age was observed across highly and most deprived urban areas (3U and 4U), with younger women showing higher positivity than older women (Supplementary Table 3). HPV screening results stratified by age and other sociodemographic characteristics are described in Table 3.

**Table 3:** Screening results by sociodemographic characteristics.

	Total screened	Positivity		*p*-value^2^	hrHPV positive no HPV16/18		HPV16 positive		HPV18 positive	
	*N*	*N*	%^1^		*N*	%^1^^,^^3^	*N*	%^1^^,^^3^	*N*	%^1^^,^^3^
Total	5,140	608	11.8		482	79.3	99	16.3	27	4.4
Median age		43	[36–50]	<0.001	43	[36–50]	43	[36–50]	43	[36–47]
Age groups				<0.001						
30–34 years	561	122	21.7		98	80.3	20	16.4	4	3.3
35–39 years	714	115	16.1		91	79.1	16	13.9	8	7.0
40–44 years	911	113	12.4		85	75.2	23	20.4	5	4.4
45–49 years	1,031	111	10.8		90	81.1	15	13.5	6	5.4
50–54 years	774	66	8.5		56	84.8	9	13.6	1	1.5
55–59 years	596	48	8.1		36	75.0	10	20.8	2	4.2
60–65 years	553	33	6.0		26	78.8	6	18.2	1	3.0
Medea Index^4^				0.18						
Semirural areas (1R)	84	12	14.3		8	66.7	4	33.3	0	0.0
Least deprived urban areas (1U)	305	46	15.1		41	89.1	5	10.9	0	0.0
Moderately deprived urban areas (2U)	–	–	–		–	–	–	–	–	–
Highly deprived urban areas (3U)	2,982	348	11.7		273	78.4	58	16.7	17	4.9
Most deprived urban areas (4U)	1,769	202	11.4		160	79.2	32	15.8	10	5.0
ASSIR region				0.01						
El Prat de Llobregat	2,324	246	10.6		200	81.3	36	14.6	10	4.1
Baix Llobregat-Litoral	2,816	362	12.9		282	77.9	63	17.4	17	4.7

1 Percentages correspond to row percentages. For continuous variables, IQR is used.

2 *p*-value resulting from the comparison between HPV positives and HPV negatives.

3 Percentage calculated among those HPV positives.

4 MEDEA Index for the participating municipalities included. No 2R or 2U areas were participating in the implementation pilot. *p*-value was calculated only comparing urban settings (1U, 3U, 4U).

### Triage cytology results

All HPV-positive women were referred to gynecologic primary care centres for triage cytology, with samples collected by a healthcare professional. This follow-up was completed in 98.7% of positive cases (*N* = 600) up to the end of April 2024 (4 months after pilot participation was completed).

Triage cytology results by HPV genotype are presented in Figure 5 and in Supplementary Table 4. The most frequent cytological abnormalities were ASC-US and LSIL, each occurring in approximately 15% of the HPV-positive cases (Supplementary Table 4). When cytological results were grouped into low-grade (ASCUS and LSIL) and high-grade lesions (HSIL, ASC-H and AGC-NOS), statistically significant differences were observed across age groups, with low-grade lesions being more frequent in younger women (*p-value = 0.008; data not shown*).

HPV16 was associated with the highest proportion of cytological abnormalities, with 49.5% of women showing a positive triage cytology result. It also had the highest proportion (20%) of high-grade lesions (HSIL, ASC-H, AGC-NOS) compared to HPV18 (7.4%) and other hr-HPV infections (6.8%) (Supplementary Table 4).

### Colposcopy referrals, biopsies and conization results

Among the 600 available triage cytology results, 41.7% of women (*N* = 250) required colposcopy referral as per protocol. Of these, 61.2% were classified as moderate-high risk (*N* = 153), 38.0% as high risk (*N* = 95), and 0.8% (*N* = 2) as very-high risk colposcopies. Within 7 months after the expected date of performance according to protocol, a total of 243 colposcopies were performed, resulting in a colposcopy referral protocol compliance of 97.2%.

Following the initial colposcopy (*N* = 243), a total of 137 biopsies (56.4%) were performed after a period of 7 months (Figure 5). Overall, the most common histological result was normal (48.9%, *N* = 67), followed by LSIL/CIN1 (32.1%, *N* = 44), HSIL/CIN2-3 (15.3%, *N* = 21) and AIS (0.7%, *N* = 1). Four samples were suboptimal for pathological diagnosis. Additionally, 10 colposcopies and biopsies were performed outside protocol recommendations, which advised co-testing after 1 year. All procedures ruled out a pathological result (Figure 5).

The positive predictive value (PPV) of referral for colposcopy was 8.4%. The overall detection rate of CIN2+ among HPV-positive women was 3.6% (22/608), while among those with HPV16, the detection rate was notably higher at 13.1% (13/99).

Among those 22 women with histological confirmation of HSIL/CIN2+ at biopsy, 21 women (95.4%) subsequently underwent conization and one woman opted for clinical surveillance due to childbearing wish. Conization confirmed one case of AIS (4.8%), as well as 16 HSIL/CIN2-3 lesions (76.2%). In one case the conization yielded an LSIL/CIN1 lesion and in two cases the result was negative for intraepithelial lesions or malignancy. In one case, the result is not available as it was performed in the private sector.

### HPV self-sampling screening and follow-up efficiency

When considering the total screened population (*N* = 5,140), the number needed to screen (NNS) to detect one CIN2+ case was 234. This means that 234 women needed to be screened to detect one case of CIN2+, highlighting the overall effectiveness of the screening strategy.

The overall detection rate of CIN2+ among HPV-positive women was 3.6% (22/608), resulting in a number needed to follow-up (NNF) of 28, indicating that 28 HPV-positive women required follow-up to detect one case of CIN2+. If considering the HPV16 women, the NNF decreases to 8, being thus 8 HPV16 women requiring follow-up to detect one case of CIN2+, while the NNF for other hr-HPV cases rises to 54.

## Discussion

This implementation pilot supports home-based HPV self-sampling as an effective primary screening strategy for women regularly attending cervical cancer screening. Findings show high self-sampling participation (80.9%) and engagement across all age groups. The active involvement of primary care providers, midwives, and community pharmacies, combined with an SMS-based invitation and reminder system coordinated by a dedicated screening office, played a crucial role in maximizing participation and ensuring follow-up. A high return rate for self-collected samples (94.0%) was achieved, along with strong compliance with triage cytology (98.7%) and colposcopy referrals (97.2%), ensuring timely management of HPV-positive cases and the prompt treatment of high-grade cervical lesions. The study also reinforces the clinical value of HPV genotype-specific risk stratification in our screening setting, confirming the different positive predictive values associated with combinations of results and how this stratification helps to prioritize and optimize clinical pathways.

Although we were working with a population highly engaged in cervical cancer screening, community pharmacies and primary care midwives played essential roles in outreach, participation, and follow-up. Our findings support both the feasibility of this model in our setting and its potential adaptability and applicability to other healthcare systems. In contrast to our approach, established screening programs such as Australia’s—where self-sampling requires a provider’s order and is performed in clinical setting—have reported a preference for self-sampling of 40.4% and a six-month colposcopy adherence rate of 81.3% (27). Similarly, the English model, which focuses on non-attenders and relies on in-person consultations, has reported a self-sampling uptake of 55.9% (28). These differences highlight the advantages of our strategy, with pharmacies facilitating participation by addressing barriers to self-sampling and midwives ensuring follow-up after screening positive results, achieving comparable outcomes without requiring direct provider involvement in the primary testing phase (29). The success of pharmacy-based distribution aligns with studies showing a preference for pharmacy-based kit collection (15), where extended hours, proximity, and a trusted environment helped overcome logistical and psychological barriers. Pharmacist counseling increased confidence in self-sampling and self-efficacy, contributing to a high return rate (94.0%), surpassing mail-to-all strategies, where unreturned kits remain a challenge (30, 31). Additionally, this approach reduced the environmental impact associated with mailed self-sampling programs, another strategy piloted in England (28, 32). Pharmacy-based distribution model success may vary according to setting and the attributions of the pharmacy. Our findings suggest that pharmacist engagement and their role as community health agents (33) are key determinants of the success of this model, and reinforcing the need for trainings programs, such as those designed in our program (34). Our pilot also incorporated complementary studies on the impact of various communication strategies, refining invitations and reminders to optimize engagement (35, 36). Results from these studies informed adjustments that improved participation, with SMS reminders significantly increasing participation (35, 36). This underscores the potential for mobile health solutions and telemedicine follow-up in maximizing preventive healthcare efforts (37).

Participation in self-sampling increased significantly by age, with older women being more likely to participate than their younger counterparts. This finding is particularly noteworthy as older women have historically demonstrated lower participation rates in cytology-based screening (38, 39). However, a recent study in Catalonia found that self-sampling was highly preferred among older age groups (15), suggesting that this strategy may help overcome age-related barriers to screening, which in our specific context may be explained by the accessibility and convenience of visiting pharmacies given the long-standing pharmacy-based colorectal cancer screening program which targets women over 50 years (40, 41). Conversely, higher cervical screening participation among younger women has traditionally been linked to more frequent gynecological visits for family planning purposes (42), which may explain their stronger preference for clinician-collected samples and the lower self-sampling uptake observed in our study. This lower uptake among younger women may also be influenced by cultural and demographic factors. For example, in Spain, approximately 35% of women aged 30–44 are migrants (43), a population group that often faces multiple barriers to preventive healthcare, including language, administrative, and socioeconomic challenges. A similar age-related pattern has been observed in Australia’s self-sampling screening program, where uptake increases with age and peaks among women aged 70–74, with 47% of women opting for self-sampling (27). In contrast, the Dutch cervical cancer screening program has reported higher self-sampling acceptability among younger women (6). This trend has been attributed to the Dutch model’s use of mailed self-sampling kits to eligible women, which reduces logistical barriers and better accommodates younger women’s competing priorities, such as work and childcare responsibilities (6). Further research is needed to better understand these intersecting factors and to design tailored strategies that address age and context-specific barriers to self-sampling.

Our findings indicate high acceptance of home-based self-sampling among regular attendees, supporting its integration into organized programs while maintaining clinician-based options to maximize coverage. One modality does not have to detract from the other. Ultimately, it is participation, rather than screening modality, that determines program success. Ensuring accessibility and providing choice between self-sampling and clinician-based collection can optimize engagement, expand coverage, and strengthen cervical cancer prevention efforts. Notably, self-sampling acceptance by socioeconomic status in urban areas (Medea Index) exceeded 73% across all groups, with the highest participation (84.2%) in the most deprived area. This aligns with global studies that advocate for the adoption of self-sampling among hard-to-reach populations as a valuable screening tool (10, 13, 14). Our findings also suggest that pharmacy-based self-sampling distribution effectively reaches lower socioeconomic groups in our setting.

Our clinical findings align with previous research, confirming higher HPV positivity among younger women and the strong association of HPV16 with high-grade cytological abnormalities and HSIL/CIN2+ detection. The overall hrHPV positivity rate (11.8%) is consistent with national studies (~12%) (44), and similar to other European countries (45–47).

A major challenge in HPV self-sampling implementation, as highlighted by the IARC guidelines, is ensuring adequate triage and follow-up compliance, as loss to follow-up can significantly reduce program effectiveness (9). Our approach achieved remarkably high adherence to cytological triage (98.7%) and compliance with colposcopy referral (97.2%), demonstrating the effectiveness of a structured implementation strategy that integrates self-sampling within primary care workflows. Midwives played a key role in ensuring triage attendance by directly communicating results by phone, while the screening coordination office ensured protocol compliance through continuous monitoring and coordination with gynecologic primary care and referral hospitals. The protocol-established turnaround times (20) were successfully met, facilitating timely follow-up for HPV-positive women and validating the approach for the future population-based program. CIN2+ detection rates (3.6% overall, 13.1% in HPV16 infections) were comparable to international studies, reinforcing the value of genotype-specific risk stratification and risk-adapted follow-up pathways (48–50).

These findings have several potential policy implications, particularly in the context of the ongoing reforms in cervical cancer screening programs across Spain. The evidence generated by this study supports the transition towards a fully organized, population-based screening program in the region, aligned with Spanish regulations that require the entire eligible population to be actively invited to cervical cancer screening by 2029 (51). The high screening uptake observed among women over the age of 55 and from lower socioeconomic backgrounds suggests that self-sampling HPV screening can overcome structural barriers and facilitate the inclusion of underscreened groups in Spain (38). Expanding the program further could potentially help reduce cervical cancer incidence in the region, as international evidence shows that long-standing population-based screening programs—such as those in the Nordic countries—have led to significant declines in cervical cancer incidence (52). The demonstrated feasibility and high adherence rates indicate that integrating HPV self-sampling with pharmacy-based distribution of screening kits, as well as midwife-led follow-up offers a scalable model to enhance participation and reduce loss to follow-up. Therefore, investing in the training and engagement of community pharmacies and primary care midwives in program as key stakeholders is crucial for successful program delivery.

However, barriers such as differences in population engagement between opportunistic and fully population-based settings must be acknowledged. Thus, a limitation of this study is that its findings may not be fully generalizable to population-based screening programs, as it was conducted in an opportunistic screening setting where women actively sought screening. Consequently, in such context, self-sampling acceptability and follow-up compliance among those with HPV detected may be overestimated compared to organized, population-based programs that invite all eligible women. In the general population, awareness of the importance of screening and appropriate adherence to follow-up may be lower, potentially leading to reduced engagement in follow-up care. Conversely, population-based programs have a broader reach and may achieve higher detection rates of high-grade lesions, along with a greater positive predictive value for colposcopy referrals. This could enhance the overall program effectiveness and potentially result in outcomes that differ from those observed in our study (53). From an equity perspective, analyses of European screening programs have shown that the type of screening program (opportunistic versus population-based) accounts for 13.6% of the observed inequalities in screening participation (54). These findings suggest that a population-based approach could further reduce disparities compared to those observed in this study.

Furthermore, participation rates in population-based programs tend to be lower due to challenges in reaching all eligible women, including those who are underscreened or hard to reach. Therefore, targeted outreach and culturally sensitive communication strategies will be essential to replicate these participation rates in a broader population. Similarly, although SMS-based reminder system and pharmacy-based distribution offer alternative pathways that may address some of the barriers to screening may require adaptation to other contexts. In this sense, future research should explore barriers to self-sampling uptake, including reasons for refusal among women who collected but did not use the self-sampling device and those who declined participation altogether. Understanding these factors and nuances will be crucial for maximizing acceptability, participation, coverage and equity in a population-based approach.

Additionally, data availability gaps identified during the pilot indicated areas for further improvement. For example, data on past screenings was incomplete and thus could not be incorporated in the present analysis, despite its relevance as a key risk determinant. Moreover, the dataset lacked sociodemographic information needed to identify ethnic, migrant, or minority groups, which are important for detecting potential inequalities in screening participation. Enhancing data completeness and accuracy will be essential for improving future evaluations of the program. Moreover, the short follow-up period limits the assessment of long-term screening outcomes, including the detection of HSIL/CIN2+ cases in women under one-year follow-up with co-testing, as well as the long-term program impact.

### Future directions

Future research should move beyond merely identifying barriers to screening participation by thoroughly investigating the underlying factors driving these differences. Ongoing qualitative studies within this population are currently being conducted. Moreover, successful implementation depends not only on the program’s effectiveness but also on its long-term sustainability, including economic viability. To this end, a short-term budget impact analysis from a national health system perspective, based on data from this pilot is currently underway. These economic evaluations, along with the findings presented in this article, will provide policymakers with critical evidence to guide informed decisions regarding program scale-up and resource allocation. Given the importance of evaluating participation among migrant and minority groups in screening programs, future research should prioritize the systematic collection of variables that identify individuals from these populations. This is essential for assessing equity in screening participation and ensuring that underserved groups are effectively reached. Furthermore, future work should continuously investigate short-, mid-and long-term screening outcomes, cost-effectiveness, and patient-reported experiences to refine screening protocols and optimize implementation strategies, ensuring the program’s effectiveness and sustainability over time.

## Conclusion

This pilot study has been instrumental in validating circuits, workflows, and protocols, laying the foundation for the transition to a population-based cervical cancer screening program using home-based self-sampling in Catalonia. With a population-based pilot phase launched in 2024 and full-scale implementation set for 2025, these findings provide a strong basis for scaling up the program in our region and may serve as a reference model for other regions considering similar transitions. The combination of coordinated invitation and reminder strategies via SMS, pharmacy-based kit distribution, and dedicated follow-up through gynecologic primary care ensured an efficient, high-adherence screening model, facilitating timely management of HPV-positive cases while promoting equitable access. Beyond its regional impact, this study adds to the growing body of evidence supporting self-sampling integration into national cervical cancer prevention strategies. It offers valuable insights to policymakers and public health leaders seeking to expand self-sampling as a scalable and sustainable strategy for improving access, participation, and early detection of cervical cancer.

## Data Availability

The raw data supporting the conclusions of this article will be made available by the authors, without undue reservation.

## Footnotes

^1^ https://canalsalut.gencat.cat/pccu1

^2^ https://canalsalut.gencat.cat/pilot-automostra

^3^ https://scientiasalut.gencat.cat/handle/11351/10477.2

^4^ https://scientiasalut.gencat.cat/handle/11351/10788

^5^La Meva Salut, https://lamevasalut.gencat.cat/

## Group members of Catalan Cervical Cancer Screening Program working group

Laia Bruni, Ramon Escuriet, Josep Alfons Espinàs, Joan Carlos Fajardo, David Frigola, Maria Antònia Llopis, Cristina Martínez, Sandra Martínez, Paula Peremiquel-Trillas, Assumpta Ricart.
