# Supplementary material for: Building the foundations for an organized population-based cervical cancer screening program with primary HPV self-sampling in Catalonia, Spain: findings from a pilot implementation study
Source: Front Med (Lausanne). 2025 Aug 4;12:1580665. doi: 10.3389/fmed.2025.1580665 (PMC12358958; doi:10.3389/fmed.2025.1580665)
Supplement: Supplementary file 1 [file Data_Sheet_1.docx]

Building the foundations for an organized population-based cervical cancer screening program with primary HPV self-sampling in Catalonia, Spain: findings from a pilot implementation study

Supplementary Material

# Supplementary Data

No supplementary data included.

# Supplementary Figures and Tables

## Supplementary Figures

No supplementary figures included.

## Supplementary Tables

Supplementary Table 1. The RECORD statement checklist for observational studies using routinely collected health data.

Supplementary Table 2. Participation according to Medea index in urban areas, by age group.

Supplementary Table 3. Positivity according to Medea index in urban areas, by age group.

Supplementary Table 4. Triage cytology results by HPV screening test result.

Supplementary Table 5. Triage cytology results categorized in low-risk and high-risk findings, by age group.

|  | **Item No.** | **STROBE items** | **RECORD items** | | **Location in manuscript where items are reported** |
| --- | --- | --- | --- | --- | --- |
| **Title and abstract** | | | | | |
|  | 1 | (a) Indicate the study’s design with a commonly used term in the title or the abstract (b) Provide in the abstract an informative and balanced summary of what was done and what was found | RECORD 1.1: The type of data used should be specified in the title or abstract. When possible, the name of the databases used should be included. | | Abstract |
|  |  |  | RECORD 1.2: If applicable, the geographic region and timeframe within which the study took place should be reported in the title or abstract. | | Title and abstract |
|  |  |  | RECORD 1.3: If linkage between databases was conducted for the study, this should be clearly stated in the title or abstract. | | NA |
| **Introduction** | | | | | |
| Background rationale | 2 | Explain the scientific background and rationale for the investigation being reported | | | Introduction; lines 68-89 |
| Objectives | 3 | State specific objectives, including any prespecified hypotheses | | | Introduction; lines 100-103 |
| **Methods** | | | | | |
| Study Design | 4 | Present key elements of study design early in the paper | | | Materials and Methods; lines 105-230 |
| Setting | 5 | Describe the setting, locations, and relevant dates, including periods of recruitment, exposure, follow-up, and data collection | | | Materials and Methods; lines 105-211 |
| Participants | 6 | *(a) Cohort study* - Give the eligibility criteria, and the sources and methods of selection of participants. Describe methods of follow-up | RECORD 6.1: The methods of study population selection (such as codes or algorithms used to identify subjects) should be listed in detail. If this is not possible, an explanation should be provided. | | Materials and Methods; lines 126-188 |
|  |  | *Cross-sectional study* - Give the eligibility criteria, and the sources and methods of selection of participants | RECORD 6.2: Any validation studies of the codes or algorithms used to select the population should be referenced. If validation was conducted for this study and not published elsewhere, detailed methods and results should be provided. | | NA |
|  |  | *(b) Cohort study* - For matched studies, give matching criteria and number of exposed and unexposed | RECORD 6.3: If the study involved linkage of databases, consider use of a flow diagram or other graphical display to demonstrate the data linkage process, including the number of individuals with linked data at each stage. | | NA |
| Variables | 7 | Clearly define all outcomes, exposures, predictors, potential confounders, and effect modifiers. Give diagnostic criteria, if applicable. | RECORD 7.1: A complete list of codes and algorithms used to classify exposures, outcomes, confounders, and effect modifiers should be provided. If these cannot be reported, an explanation should be provided. | | Materials and Methods; lines 189-196 and 197-211 |
| Data sources/ measurement | 8 | For each variable of interest, give sources of data and details of methods of assessment (measurement). | | | Materials and Methods; lines 197-211 |
|  |  | Describe comparability of assessment methods if there is more than one group | | | NA |
| Bias | 9 | Describe any efforts to address potential sources of bias | | | NA |
| Study size | 10 | Explain how the study size was arrived at | | | NA |
| Quantitative variables | 11 | Explain how quantitative variables were handled in the analyses. If applicable, describe which groupings were chosen, and why | | | Materials and Methods; lines 212-221 |
| Statistical methods | 12 | (a) Describe all statistical methods, including those used to control for confounding | | | Materials and Methods; lines 212-221 |
|  |  | (b) Describe any methods used to examine subgroups and interactions | | | Materials and Methods; lines 212-221 |
|  |  | (c) Explain how missing data were addressed | | | NA |
|  |  | (d) Cohort study - If applicable, explain how loss to follow-up was addressed | | | NA |
|  |  | (e) Describe any sensitivity analyses | | | NA |
| Data access and cleaning methods |  | .. | RECORD 12.1: Authors should describe the extent to which the investigators had access to the database population used to create the study population. | | Materials and Methods; lines 198-211 |
|  |  |  | RECORD 12.2: Authors should provide information on the data cleaning methods used in the study. | | Materials and Methods; lines 198-211 |
| Linkage |  | .. | RECORD 12.3: State whether the study included person-level, institutional-level, or other data linkage across two or more databases. The methods of linkage and methods of linkage quality evaluation should be provided. | | Materials and Methods; lines 198-211 |
| **Results** | | | | | |
| Participants | 13 | (a) Report the numbers of individuals at each stage of the study (e.g., numbers potentially eligible, examined for eligibility, confirmed eligible, included in the study, completing follow-up, and analysed) | RECORD 13.1: Describe in detail the selection of the persons included in the study (i.e., study population selection) including filtering based on data quality, data availability and linkage. The selection of included persons can be described in the text and/or by means of the study flow diagram. | | Results; lines 233-241 |
|  |  | (b) Give reasons for non-participation at each stage. | | | Results; Figure 3 |
|  |  | (c) Consider use of a flow diagram | | | Results; Figure 3 |
| Descriptive data | 14 | (a) Give characteristics of study participants (e.g., demographic, clinical, social) and information on exposures and potential confounders | | | Results; lines 233-241 and 286-327. |
|  |  | (b) Indicate the number of participants with missing data for each variable of interest | | | Results; lines 233-255 and 286-327. |
|  |  | (c) Cohort study - summarise follow-up time (e.g., average and total amount) | | | Results; lines 233-255 and 286-327. |
| Outcome data | 15 | *Cohort study* - Report numbers of outcome events or summary measures over time | | | Figure 5 |
| Main results | 16 | (a) Give unadjusted estimates and, if applicable, confounder-adjusted estimates and their precision (e.g., 95% confidence interval). Make clear which confounders were adjusted for and why they were included | | | Results; lines 242-336 |
|  |  | (b) Report category boundaries when continuous variables were categorized | | | Results; tables and supplementary tables. |
|  |  | (c) If relevant, consider translating estimates of relative risk into absolute risk for a meaningful time period | | | NA |
| Other analyses | 17 | Report other analyses done—e.g., analyses of subgroups and interactions, and sensitivity analyses | | | Supplementary Tables 2 & 3 |
| **Discussion** | | | | | |
| Key results | 18 | Summarise key results with reference to study objectives | | | Discussion; lines 338-370 |
| Limitations | 19 | Discuss limitations of the study, taking into account sources of potential bias or imprecision. Discuss both direction and magnitude of any potential bias | | RECORD 19.1: Discuss the implications of using data that were not created or collected to answer the specific research question(s). Include discussion of misclassification bias, unmeasured confounding, missing data, and changing eligibility over time, as they pertain to the study being reported. | Discussion; lines 407-421 |
| Interpretation | 20 | Give a cautious overall interpretation of results considering objectives, limitations, multiplicity of analyses, results from similar studies, and other relevant evidence | |  | Discussion; lines 371-406 |
| Generalisability | 21 | Discuss the generalisability (external validity) of the study results | |  | Discussion; lines 407-421 |
| **Other Information** | | | | | |
| Funding | 22 | Give the source of funding and the role of the funders for the present study and, if applicable, for the original study on which the present article is based | | | Funding section |
| Accessibility of protocol, raw data, and programming code |  | .. | RECORD 22.1: Authors should provide information on how to access any supplemental information such as the study protocol, raw data, or programming code. | | Data Availability Statement section |

**Supplementary Table 1. The RECORD statement checklist for observational studies using routinely collected health data.**

|  | **Least deprived urban areas (1U)** | | | | **Moderately deprived urban areas (2U)** | **Highly deprived urban areas (3U)** | | | | **Most deprived urban areas (4U)** | | | |
| --- | --- | --- | --- | --- | --- | --- | --- | --- | --- | --- | --- | --- | --- |
|  | **Invited** | **Participants** | **Participation** | ***p-value^2^*** | **-** | **Invited** | **Participants** | **Participation** | ***p-value^2^*** | **Invited** | **Participants** | **Participation** | ***p-value^2^*** |
|  | **N** | **N** | ***%^1^*** |  | **-** | **N** | **N** | **%*^1^*** |  | **N** | **N** | **%*^1^*** |  |
| **Total** | 416 | 305 | *73.3* |  | - | 3,731 | 2,982 | *79.9* |  | 2,100 | 1,769 | *84.2* |  |
| **Age groups** |  |  |  | *0.66* | - |  |  |  | *<0.001* |  |  |  | *<0.001* |
| ***30-34 years*** | 60 | 40 | *66.7* |  | - | 473 | 337 | *71.2* |  | 238 | 180 | *75.6* |  |
| ***35-39 years*** | 65 | 49 | *75.4* |  | - | 534 | 401 | *75.1* |  | 299 | 254 | *84.9* |  |
| ***40-44 years*** | 91 | 64 | *70.3* |  | - | 656 | 521 | *79.4* |  | 367 | 301 | *82.0* |  |
| ***45-49 years*** | 75 | 54 | *72.0* |  | - | 706 | 583 | *82.6* |  | 445 | 377 | *84.7* |  |
| ***50-54 years*** | 56 | 44 | *78.6* |  | - | 582 | 479 | *82.3* |  | 289 | 241 | *83.4* |  |
| ***55-59 years*** | 31 | 23 | *74.2* |  | - | 418 | 353 | *84.4* |  | 231 | 209 | *90.5* |  |
| ***60-65 years*** | 38 | 31 | *81.6* |  | - | 362 | 308 | *85.1* |  | 231 | 207 | *89.6* |  |

**Supplementary Table 2. Participation according to Medea index in urban areas, by age group.** No 2U areas were participating in the implementation pilot. ^1^ Percentages correspond to column percentages. ^2^ P-value resulting from the comparison between participants and non-participants.

|  | **Least deprived urban areas (1U)** | | | | **Moderately deprived urban areas (2U)** | **Highly deprived urban areas (3U)** | | | | **Most deprived urban areas (4U)** | | | |
| --- | --- | --- | --- | --- | --- | --- | --- | --- | --- | --- | --- | --- | --- |
|  | **Participants** | **Positive** | **Positivity** | ***p-value^2^*** | **-** | **Participants** | **Positive** | **Positivity** | ***p-value^2^*** | **Participants** | **Positive** | **Positivity** | ***p-value^2^*** |
|  | **N** | **N** | **%*^1^*** |  | **-** | **N** | **N** | **%*^1^*** |  | **N** | **N** | **%*^1^*** |  |
| **Total** | 305 | 46 | *15.1* |  | - | 2,982 | 348 | *11.7* |  | 1,769 | 202 | *11.4* |  |
| **Age groups** |  |  |  | *0.98* | - |  |  |  | *<0.001* |  |  |  | *<0.001* |
| ***30-34 years*** | 40 | 6 | *15.0* |  | - | 337 | 76 | *22.6* |  | 180 | 38 | *21.1* |  |
| ***35-39 years*** | 49 | 9 | *18.4* |  | - | 401 | 65 | *16.2* |  | 254 | 38 | *15.0* |  |
| ***40-44 years*** | 64 | 9 | *14.1* |  | - | 521 | 63 | *12.1* |  | 301 | 38 | *12.6* |  |
| ***45-49 years*** | 54 | 9 | *16.7* |  | - | 583 | 62 | *10.6* |  | 377 | 38 | *10.1* |  |
| ***50-54 years*** | 44 | 7 | *15.9* |  | - | 479 | 35 | *7.3* |  | 241 | 22 | *9.1* |  |
| ***55-59 years*** | 23 | 3 | *13.0* |  | - | 353 | 30 | *8.5* |  | 209 | 15 | *7.2* |  |
| ***60-65 years*** | 31 | 3 | *9.7* |  | - | 308 | 17 | *5.5* |  | 207 | 13 | *6.3* |  |

**Supplementary Table 3. Positivity according to Medea index in urban areas, by age group.** No 2U areas were participating in the implementation pilot. ^1^Percentages correspond to column percentages.^2^P-value resulting from the comparison between HPV positives and HPV negatives.

|  | **HPV positive** | | **HPV16 positive** | | | **HPV18 positive** | | | **Other hr-HPV positive** | | |
| --- | --- | --- | --- | --- | --- | --- | --- | --- | --- | --- | --- |
|  | **N** | **%^1^** | **N** | **%^1^** | **%^2^** | **N** | **%^1^** | **%^2^** | **N** | **%^1^** | **%^2^** |
| **Total^2^** | 608 | *100.0* | 99 | *50.5* | *16.3* | 27 | *59.3* | *4.4* | 482 | *62.9* | *79.3* |
| **Triage cytology results** |  |  |  |  |  |  |  |  |  |  |  |
| ***NILM*** | 369 | *60.7* | 50 | *13.1* | *13.6* | 16 | *11.1* | *4.3* | 303 | *15.8* | *82.1* |
| ***ASC-US*** | 92 | *15.1* | 13 | *14.1* | *14.1* | 3 | *18.5* | *3.3* | 76 | *15.6* | *82.6* |
| ***LSIL*** | 94 | *15.5* | 14 | *9.1* | *14.9* | 5 | *3.7* | *5.3* | 75 | *2.1* | *79.8* |
| ***HSIL*** | 20 | *3.3* | 9 | *11.1* | *45.0* | 1 | *0.0* | *5.0* | 10 | *2.3* | *50.0* |
| ***ASC-H*** | 22 | *3.6* | 11 | *0.0* | *50.0* | 0 | *3.7* | *0.0* | 11 | *0.4* | *50.0* |
| ***AGC*** | 3 | *0.5* | 0 | *2.0* | *0.0* | 1 | *3.7* | *33.3* | 2 | *1.0* | *66.7* |
| ***Not performed^3^*** | 8 | *1.3* | 2 | *100.0* | *25.0* | 1 | *100.0* | *12.5* | 5 | *100.0* | *62.5* |

**Supplementary Table 4. Triage cytology results by HPV screening test result.** ^1^Corresponds to column percentage. ^2^Percentage according to each triage cytology result (row percentage). ^3^Triage cytologies not performed seven months after the expected date of performance according to protocol. AGC: atypical glandular cells; ASC-H: atypical squamous cells, cannot exclude high-grade squamous intraepithelial lesion; ASC-US: atypical squamous cells of undetermined significance; HPV: Human papillomavirus; hrHPV: High-risk HPV; HSIL: High-grade squamous intraepithelial lesion; LSIL: Low-grade squamous intraepithelial lesion, NILM: Negative for intraepithelial lesion or malignancy.

|  | **Low-risk cytologic lesions**  ***ASC-US and LSIL*** | | **High risk cytologic lesions**  ***ASC-H, HSIL and AGC*** | | ***p-value^2^*** |
| --- | --- | --- | --- | --- | --- |
|  | **N** | **%^1^** | **N** | **%^1^** |  |
| **Total^2^** | 186 | *80.5* | 45 | *19.5* |  |
| **Age groups** |  |  |  |  | *0.008* |
| ***30-34 years*** | 44 | *81.5* | 10 | *18.5* |  |
| ***35-39 years*** | 39 | *79.6* | 10 | *20.4* |  |
| ***40-44 years*** | 32 | *82.1* | 7 | *17.9* |  |
| ***45-49 years*** | 42 | *93.3* | 3 | *6.7* |  |
| ***50-54 years*** | 11 | *55.0* | 9 | *45.0* |  |
| ***55-59 years*** | 13 | *86.7* | 2 | *13.3* |  |
| ***60-65 years*** | 5 | *55.6* | 4 | *44.4* |  |

**Supplementary Table 5. Triage cytology results categorized in low-risk and high-risk findings, by age group.**^1^Percentage according to the total number of cytologic abnormalities detected (row percentage). ^2^P-value was calculated comparing low-risk and high-risk cytologic lesions. AGC: atypical glandular cells; ASC-H: atypical squamous cells, cannot exclude high-grade squamous intraepithelial lesion; ASC-US: atypical squamous cells of undetermined significance; HPV: Human papillomavirus; hrHPV: High-risk HPV; HSIL: High-grade squamous intraepithelial lesion; LSIL: Low-grade squamous intraepithelial lesion, NILM: Negative for intraepithelial lesion or malignancy.
